# The use of genomic coancestry matrices in the optimisation of contributions to maintain genetic diversity at specific regions of the genome

**DOI:** 10.1186/s12711-015-0172-y

**Published:** 2016-01-13

**Authors:** Fernando Gómez-Romano, Beatriz Villanueva, Jesús Fernández, John A. Woolliams, Ricardo Pong-Wong

**Affiliations:** 1 Departamento de Mejora Genética Animal, INIA, Madrid, Spain; 2 The Roslin Institute and the R(D)SVS, University of Edinburgh, Edinburgh, UK

## Abstract

**Background:**

Optimal contribution methods have proved to be very efficient for controlling the rates at which coancestry and inbreeding increase and therefore, for maintaining genetic diversity. These methods have usually relied on pedigree information for estimating genetic relationships between animals. However, with the large amount of genomic information now available such as high-density single nucleotide polymorphism (SNP) chips that contain thousands of SNPs, it becomes possible to calculate more accurate estimates of relationships and to target specific regions in the genome where there is a particular interest in maximising genetic diversity. The objective of this study was to investigate the effectiveness of using genomic coancestry matrices for: (1) minimising the loss of genetic variability at specific genomic regions while restricting the overall loss in the rest of the genome; or (2) maximising the overall genetic diversity while restricting the loss of diversity at specific genomic regions.

**Results:**

Our study shows that the use of genomic coancestry was very successful at minimising the loss of diversity and outperformed the use of pedigree-based coancestry (genetic diversity even increased in some scenarios). The results also show that genomic information allows a targeted optimisation to maintain diversity at specific genomic regions, whether they are linked or not. The level of variability maintained increased when the targeted regions were closely linked. However, such targeted management leads to an important loss of diversity in the rest of the genome and, thus, it is necessary to take further actions to constrain this loss. Optimal contribution methods also proved to be effective at restricting the loss of diversity in the rest of the genome, although the resulting rate of coancestry was higher than the constraint imposed.

**Conclusions:**

The use of genomic matrices when optimising contributions permits the control of genetic diversity and inbreeding at specific regions of the genome through the minimisation of partial genomic coancestry matrices. The formula used to predict coancestry in the next generation produces biased results and therefore it is necessary to refine the theory of genetic contributions when genomic matrices are used to optimise contributions.

## Background

It is generally accepted that control of the rate of coancestry provides a general framework to manage genetic variability. Optimal contribution (OC) methods [1, 2] permit the determination of the number of offspring that each breeding candidate should produce to minimise coancestry. These methods were initially developed based on a pedigree-based relationship matrix (**A**) that represents expected relationships assuming neutrality and does not take into account variation due to Mendelian sampling. Thus, although its use has proved to be efficient to manage genetic diversity, it has some limitations. For instance, individuals from the same (full-sib) family inherit different sets of alleles but they are assumed to be equally related. In addition, since matrix **A** does not consider differences between genomic regions, optimisation of contributions will, on average, control the rate of coancestry to the chosen value, but some genomic regions may have substantially higher rates than desired.

The management of genetic diversity can be improved if matrix **A** is replaced by a realised relationship matrix that is calculated by taking into account variation in the level of relationship between animals of the same family and variation between genomic regions [3]. Because of the availability of high-density single nucleotide polymorphism (SNP) chips, it is now possible to calculate such realised relationship matrices. Genotypes for hundreds or thousands of SNPs across the genome are now commonly used to calculate relationship matrices for many species (e.g., [3–5]). These matrices have proved to satisfactorily manage global genetic diversity and outperform pedigree-based relationship matrices [6–8]. Marker-based relationship matrices can also be used to minimise loss of variability at specific regions of the genome, which is useful for certain genomic regions. For example, for regions that harbour loci involved in general resistance to disease [e.g. the major histocompatibility complex (MHC)] a high level of genetic diversity is desirable to ensure that the population can deal with potential new disease challenges. This is also the case for regions that are associated with inbreeding depression for fitness traits [9, 10]. In addition, evolutionary forces such as genetic drift and selection can lead to genomic regions that have substantially less genetic variation than other regions. In fact, several studies have reported that variation in genetic diversity between regions could be quite large (e.g., [11–13]). Thus, conservation programmes could be more efficient if approaches to maintain genetic diversity focussed on some regions of the genome (regardless of whether they contain known genes of interest) rather than on the whole genome. However, such approaches require that constraints on coancestry are imposed on the rest of the genome. Otherwise, rates of coancestry, inbreeding and loss of variability could become high in regions that are positioned away from the region that was targeted for minimisation [14].

The objective of this study was to assess, through computer simulations, the effectiveness of using dense SNP panels when contributions are optimised to: (1) minimise the loss of genetic variability at specific genomic regions while restricting the overall loss of diversity in the rest of the genome; or (2) maximise the overall genetic diversity while restricting the loss of diversity at specific genomic regions.

## Methods

### Optimisation of contributions to minimise the loss of genetic diversity

Assume a set of *N* breeding candidates and **c** the vector of genetic contributions of the candidates to the next (offspring) generation. These contributions represent the fraction of the genetic material that each candidate contributes to the gene pool of the next generation. In diploid species, each sex contributes half of the gene pool, so the genetic contribution of a given candidate ranges from 0 to 0.5. Note that *c*_*i*_ = 0 indicates that the candidate *i* has no offspring and *c*_*i*_ = 0.5 indicates that all offspring are fathered (or mothered) by *i*. Let **s** and **d** be vectors of indicators of the sex of the candidates, with *s*_*i*_ = 1 if candidate *i* is male and 0 if it is female, and **d** = **1** − **s**.

#### Optimisation problem 1

When the main breeding objective is to minimise the loss of genetic diversity, genetic contributions of candidates are optimised by minimising the expected average level of coancestry in the offspring generation. Hence, the OC problem can be formulated as:

1a $$ {\text{Minimise}}\quad {\mathbf{c}}^{\prime }{\mathbf{Gc}}, $$

1b $$ {\text{subject to}}\quad {\mathbf{c}}^{\prime }{\mathbf{s}} = 0. 5, $$

1c $$ {\mathbf{c}}^{\prime }{\mathbf{d}} = 0. 5, $$

1d $$ c_{i} \ge 0, $$

where **G** is the coancestry matrix containing coefficients of coancestry between all candidates in the population. Note that this differs from the formulations of Meuwissen [1], Grundy et al. [2] and Pong-Wong and Woolliams [15], who used the numerator relationship matrix **A** which is twice **G** (i.e., **G** = ½**A**). The constraints (1b–d) are imposed in order to keep the solution for **c** within the valid range.

Matrix **G** can be computed from pedigree or molecular data. With the availability of dense SNP genotypes, it is also possible to obtain a **G** matrix for specific regions of the genome. Hence, the optimisation problem can be implemented to minimise the loss of diversity across the whole genome or at specific regions of the genome by using the appropriate **G** matrix (see below).

#### Optimisation problem 2

While keeping the objective of minimising the loss of diversity (across the genome or at specific regions), the optimisation problem can be refined by imposing additional constraints so that the expected level of coancestry in the offspring generation for one or more genome regions cannot be greater than a given predefined value (*k*). Hence, the OC problem can be reformulated by adding *m* additional constraints:

2a $$ {\text{Minimise}}\quad {\mathbf{c}}^{\prime }{\mathbf{Gc}}, $$

2b $$ {\text{subject to}}\quad {\mathbf{c}}^{\prime }{\mathbf{G}}_{{\mathbf{1}}} {\mathbf{c}} \le k_{ 1} , $$

$$ \begin{aligned} {\mathbf{c}}^{\prime }{\mathbf{G}}_{{\mathbf{2}}} {\mathbf{c}} &\le k_{ 2} , \hfill \\ \vdots \hfill \\ {\mathbf{c}}^{\prime }{\mathbf{G}}_{{\mathbf{m}}} {\mathbf{c}} &\le k_{\text{m}} , \hfill \\ \end{aligned} $$

2c $$ {\mathbf{c}}^{\prime }{\mathbf{s}} = 0. 5, $$

2d $$ {\mathbf{c}}^{\prime }{\mathbf{d}} = 0. 5, $$

2e $$ c_{i} \ge 0, $$

where **G** is the matrix for the part of the genome where coancestry will be minimised (overall or local) and **G**_***j***_ (*j* = 1, …, *m*) is the coancestry matrix for region *j* for which a restriction is imposed. The term *k*_*j*_ is the maximum expected level of coancestry allowed for region *j*. For a given generation, *k*_*j*_ can be calculated as *k*_*j*_ = 1 − (1 − *C*_*j*_)(1 − *f*_*j*_), where *f*_*j*_ is the average coancestry at region *j* in that generation, and *C*_*j*_ is the targeted rate of coancestry for region *j*.

The implementation of both optimisation problems was carried out using a semidefinite programming (SDP) approach as described in Pong-Wong and Woolliams [15]. In order to do so, the optimisation problems 1 and 2 were, first, reformulated as standard SDP problems and, thereafter, solved using the SDPA package [16]. Details on how they are reformulated as standard SDP problems are in the “Appendix”.

### Coancestry matrices

Different coancestry matrices were used in the optimisation of contributions. They included coancestry matrices computed from pedigree or genomic information. Genomic matrices were calculated using a large number of biallelic markers that mimicked SNPs and the allelic similarity method proposed by Nejati-Javaremi et al. [3]. For a given SNP, the allelic relationship between two individuals is $\left( {0.25} \right) \sum\nolimits_{i = 1}^{2} {\sum\nolimits_{j = 1}^{2} {\delta_{ij} } }$, where *δ*_*ij*_ is the allele sharing status, which is equal to 1 if allele *i* from the first individual is identical to allele *j* from the second individual and 0 otherwise. The genomic coancestry between two individuals is the average genomic coancestry across all genotyped SNPs in the genome (for the whole genome matrix) or in the regions of interest (for regional genomic matrices). Note that, although the realised coancestry matrix based on SNP data is more precise than the traditional pedigree-based coancestry matrix, it still represents estimates of the true relationships unless full sequences are available and used to calculate relationships.

### Simulations

Different management strategies aimed at minimising the loss of genetic diversity were compared using Monte Carlo simulations. The strategies differed in the type of information used to compute coancestries when optimising contributions. They also differed in the objective function to be minimised and the restrictions imposed during the optimisation.

The study considered populations of *N* animals (20 or 100) born per generation. The sex of the individuals was randomly assigned, with 50 % males and 50 % females. Each management scenario was replicated 100 times.

### Genetic and population models

The genetic model assumed that the genome was divided into 20 chromosomes of 1 Morgan each. Each chromosome had *n*_*loci*_ biallelic loci equally spaced. The genotypes of *n*_*loci*_/2 of the loci (those located at alternate positions) were assumed to be known and they were used to create the genomic matrices required for the optimisation of contributions. Thus, these *n*_*loci*_/2 loci simulated per chromosome mimicked genotyped SNPs. The remaining *n*_*loci*_/2 loci per chromosome were used to assess the effect of the different management strategies on the amount of diversity maintained. Both types of loci were simulated in the same way and, as described below, differed simply in their use: marker loci were used for taking management decisions, whereas non-marker loci were used to measure true coancestry. In practice, commercial SNP chips represent a proportion of the full sequence and they are not designed to include rare SNPs and causative mutations. In this study, we assumed that the interest lies in the diversity of the non-marker loci, thus the relationships computed using the non-marker loci are referred to as the true relationships. The coancestry matrix calculated with the observed marker loci (and used in the optimisation) is assumed to be an estimate of the true coancestry matrix.

Initially, a base population in mutation-drift equilibrium was generated. This ensured the existence of linkage disequilibrium (LD) between marker and non-marker loci. Details on how the base population was created are in Gómez-Romano et al. [8]. In brief, a historical population of size *N* was simulated for 10,000 generations of random mating. The historical population was initialised assuming that alleles at the 20*n*_*loci*_ simulated loci were fixed. Two different mutation rates were considered (*μ* = 2.5 × 10^−3^ and *μ* = 2.5 × 10^−5^) in order to mimic two different degrees of LD between marker and non-marker loci. The last generation of this process was considered to be the base population (*t* = 0). In scenarios with *μ* = 2.5 × 10^−3^, *n*_*loci*_ was equal to 2000 and in scenarios with *μ* = 2.5 × 10^−5^, it was equal to 60,000. These values for *n*_*loci*_ ensured that there was a sufficient number of loci segregating at *t* = 0, i.e. at least 1200 for *μ* = 2.5 × 10^−3^ and 1300 for *μ* = 2.5 × 10^−5^ SNPs were segregating per chromosome, resulting in a total of 24,000 and 26,000 SNPs for the whole genome, respectively. Only the loci that were segregating at *t* = 0 were used for analysis.

From *t* = 0 onwards, the population was managed under different strategies for 10 generations. In each generation, the contributions of the potential parents were optimised according to the strategy used, and a generation of offspring of size *N* was generated with random mating based on optimised contributions. In turn, the offspring produced became the candidates for the next round. It should be noted that mutation rate was set to zero during these ten generations of management.

### Scenarios compared

Seven management strategies (PED, OVE, CHR, REG, OVE__reg_, CHR__ove_ and REG__ove_) were considered (Table 1). Management in strategies PED, OVE, CHR and REG was based on optimisation problem 1 and differed in the coancestry (*f*) minimised (i.e., in the **G** matrix used in Eq. 1a). Strategy PED minimised pedigree-based coancestry (*f*_*p*_), OVE minimised the overall (i.e., average for all markers in the genome) genomic coancestry (*f*_*m_ove*_), CHR minimised coancestry across an entire chromosome (arbitrarily chosen to be chromosome 1) (*f*_*m_chr*_), and REG minimised the average genomic coancestry (*f*_*m_reg*_) across 10 regions of 10 cM each located on 10 different chromosomes. Since the proportion of the genome to be minimised was the same for strategies CHR and REG, CHR can be considered as a special case of REG in which all regions are located on the same chromosome. The specific locations of the 10 regions minimised under REG were randomly chosen. Strategies CHR__ove_ and REG__ove_ were based on optimisation problem 2, in which the average coancestry in specific regions of the genome was minimised while restricting the coancestry in the rest of the genome (*f*_*m_ove*-*chr*_ and *f*_*m_ove*-*reg*_ for CHR__ove_ and REG__ove_, respectively). The restriction applied to the rest of the genome was such that the intended rate of increase in *f*_*m_ove*-*chr*_ and *f*_*m_ove*-*reg*_ was either 1.0 or 0.1 % per generation. Strategy OVE__reg_ was also based on optimisation problem 2 and implied minimising the overall genomic coancestry while imposing independent restrictions (0.10 or 0.01 %) on the increase in coancestry at each of the 10 regions on different chromosomes (*f*_*m_reg*_). An additional scenario where contributions were randomly assigned (strategy RAN) was also considered for comparison.

**Table 1:** Rates of coancestry minimised and restricted for each optimisation strategy

Strategy	Minimisation	Restriction
PED	Pedigree coancestry	–
OVE	Overall genomic coancestry	–
CHR	Average genomic coancestry for chromosome 1	–
REG	Average genomic coancestry across 10 regions of 10 cM each, located on different chromosomes	–
OVE__reg_	Overall genomic coancestry	Rate of genomic coancestry for each of 10 regions of 10 cM each, located on different chromosomes
CHR__ove_	Average genomic coancestry for chromosome 1	Overall rate of genomic coancestry
REG__ove_	Average genomic coancestry across 10 regions of 10 cM each, located on different chromosomes	Overall rate of genomic coancestry

### Criteria of comparison

The rate at which genetic diversity is lost is given by the rate at which the average true coancestry increases (Δ*f*). Thus, the main criterion for comparing management strategies was the true genomic rate calculated for each generation, as well as the pedigree-based rate of coancestry. For the purpose of comparing strategies, the true relationship between individuals was assumed to be the genomic coancestry matrix computed using the non-marker loci. The number of individuals that contributed to the offspring generation and the variance of contributions were also calculated for each generation.

## Results

Table 2 shows the average rates of pedigree and true molecular coancestries for scenarios RAN, PED and OVE, when the mutation rate (*μ*) in the historical population was assumed to be either 2.5 × 10^−3^ or 2.5 × 10^−5^. These differences in mutation rate resulted in different levels of LD between adjacent SNPs at *t* = 0, i.e. for *μ* = 2.5 × 10^−3^, average LD was equal to 0.28 and 0.13 for *N* = 20 and 100, respectively and for *μ* = 2.5 × 10^−5^, it was equal to 0.40 and 0.21, respectively.

**Table 2:** Rates of pedigree and overall true genomic coancestry across generations (*t*) when applying different management strategies (RAN, PED and OVE) in populations of two different sizes (*N*) and using two mutation rates (*μ*) to create the base population

*N*	*t*	Rate of pedigree coancestry (%)						Rate of genomic coancestry (%)					
		*μ* = 2.5 × 10^−3^			*μ* = 2.5 × 10^−5^			*μ* = 2.5 × 10^−3^			*μ* = 2.5 × 10^−5^		
		RAN	PED	OVE	RAN	PED	OVE	RAN	PED	OVE	RAN	PED	OVE
20	1	2.46	1.28	2.47	2.45	1.28	2.69	2.47	1.32	0.17	2.57	1.31	0.20
	2	2.40	1.30	1.79	2.39	1.30	1.97	2.47	1.25	1.32	2.45	1.31	1.01
	3	2.44	1.30	1.73	2.43	1.30	1.89	2.34	1.24	1.29	2.30	1.32	1.08
	4	2.52	1.30	1.70	2.52	1.30	1.89	2.55	1.30	1.40	2.48	1.32	1.05
	5	2.46	1.30	1.75	2.45	1.30	1.88	2.40	1.35	1.50	2.48	1.30	1.10
	10	2.39	1.30	1.81	2.39	1.30	1.85	2.36	1.28	1.47	2.42	1.35	1.07
100	1	0.50	0.25	0.74	0.52	0.25	1.05	0.50	0.26	−0.16	0.55	0.22	−0.40
	2	0.51	0.25	0.50	0.48	0.25	0.69	0.50	0.25	0.23	0.57	0.25	−0.16
	3	0.49	0.25	0.49	0.46	0.25	0.65	0.50	0.25	0.28	0.44	0.19	−0.15
	4	0.50	0.25	0.48	0.52	0.25	0.63	0.51	0.26	0.31	0.60	0.27	−0.05
	5	0.50	0.25	0.47	0.51	0.25	0.64	0.50	0.26	0.34	0.60	0.25	−0.07
	10	0.50	0.25	0.46	0.50	0.25	0.58	0.51	0.26	0.37	0.47	0.19	−0.03

Average linkage disequilibrium between consecutive SNPs at *t* = 0 was 0.28 and 0.13 for *N* = 20 and 100, respectively when *μ* = 2.5 × 10^−3^, and 0.40 and 0.21 for *N* = 20 and 100, respectively when *μ* = 2.5 × 10^−5^

*RAN* contributions are assigned at random, *PED* contributions are optimised to minimise *f*_*p*_, *OVE* contributions are optimised to minimise *f*_*m_ove*_

As expected, across different selection scenarios and levels of mutation, the rate of coancestry (pedigree or molecular) was always higher with the smaller population size. Rates of pedigree and true genomic coancestry were similar for the strategies RAN and PED, but large differences were observed when the contributions were optimised to minimise *f*_*m_ove*_ (strategy OVE). These results clearly show that pedigree coancestry is not a good estimator of the true coancestry when genomic relationships are used in the optimisation of contributions. The performance of OVE depended on the mutation rate used to create the base population. For *μ* = 2.5 × 10^−5^, OVE substantially outperformed PED by having a much lower rate of true genomic coancestry across all generations and population sizes. Across generations, the average rate of increase of genomic coancestry (Δ*f*_*m_ove*_) was equal to 0.0132 (*N* = 20) and 0.0023 (*N* = 100) for PED versus 0.0092 (*N* = 20) and −0.0014 (*N* = 100) for OVE (negative rate means a decrease in the genomic coancestry). However, with a higher mutation rate (*μ* = 2.5 × 10^−3^), the advantage of OVE over PED in terms of lower Δ*f*_*m_ove*_ was only observed for the first generation and Δ*f*_*m_ove*_ became slightly higher under OVE than under PED in later generations. Conversely, this good performance of OVE in early generations implies that its actual level of true coancestry remained lower than with PED at later generations (data not shown). These results suggest that the level of LD in scenarios with the higher mutation rate (*μ* = 2.5 × 10^−3^) may not be sufficient for the genomic coancestry calculated with the observed SNPs (and used in optimisation) to be a good estimator of the true coancestry for unobserved loci. Hence, the remaining results will be based only on populations that were simulated assuming a mutation rate of *μ* = 2.5 × 10^−5^.

Tables 3 (*N* = 20) and 4 (*N* = 100) show the rate of genomic coancestry for the targeted regions (those where Δ*f* was minimised) and for the rest of the genome under strategies CHR and REG. The OC method was very efficient in avoiding loss of diversity in the considered regions to the point that, for most generations, genetic diversity even increased (i.e., the rate of coancestry was negative). The optimisation was more successful when all targeted regions were on the same chromosome (CHR) than when they were located on different chromosomes (REG), although the proportion of the genome for which coancestry was minimised was the same (5 %). In both scenarios (CHR and REG), the success in maintaining more diversity in specific regions had undesired consequences for the rest of the genome, where the observed Δ*f* was several folds higher than when assuming random selection or when optimisation was based on pedigree coancestry (cf. Table 2). For instance, for *N* = 20, Δ*f* for the rest of the genome with REG and CHR was respectively two and three folds higher than the observed Δ*f* with PED. For *N* = 100, these differences were five and nine folds higher, respectively. Moreover, the poor performance for the rest of the genome was related to the good performance for the regions targeted for minimisation, with CHR being the most efficient for maintaining diversity in these regions but also being the worst in losing it for the rest of the genome.

**Table 3:** Average rate of genomic coancestry in genomic regions targeted for minimising coancestry and in the rest of the genome across generations (*t*), when applying different management strategies (CHR, CHR__ove_, REG, REG__ove_) for a population of size 20

*t*	CHR	CHR__ove_		REG	REG__ove_	
		*C* = 1.0 %	*C* = 0.1 %		*C* = 1.0 %	*C* = 0.1 %
Rate of genomic coancestry at regions targeted for minimisation (%)						
1	−4.64	−4.33	−3.79	−2.18	−2.06	−1.78
2	−1.08	−1.09	−0.54	−0.39	−0.64	−0.16
3	−0.06	−0.28	−0.22	−0.33	−0.32	−0.05
4	−0.06	0.14	0.04	−0.25	0.23	0.09
5	0.30	0.22	0.14	0.10	0.25	0.23
10	0.42	0.37	0.35	0.54	0.35	0.40
Rate of genomic coancestry at the rest of genome (%)						
1	6.28	2.27	1.47	3.86	2.42	1.67
2	4.34	2.44	1.52	3.22	2.26	1.59
3	4.20	2.32	1.40	3.31	2.32	1.61
4	4.04	2.38	1.50	3.05	2.46	1.59
5	3.82	2.37	1.48	2.98	2.30	1.67
10	3.28	2.40	1.40	2.81	2.25	1.45

Two different constraints (*C*) were imposed on the rate of coancestry at the rest of the genome when applying strategies CHR__ove_ and REG__ove_

*CHR* contributions are optimised to minimise *f*_*m_chr*_, *CHR*_*_ove*_ contributions are optimised to minimise *f*_*m_chr*_ while restricting *f*_*m_ove*-*chr*_, *REG* contributions are optimised to minimise *f*_*m_reg*_, *REG*_*_ove*_ contributions are optimised to minimise *f*_*m_reg*_ while restricting *f*_*m_ove*-*reg*_

In order to control this detrimental effect, an additional constraint was imposed to restrict the excessive loss of diversity across the rest of the genome (strategies CHR__ove_ and REG__ove_). The inclusion of such constraints succeeded in substantially reducing the rate of increase in coancestry, but the realised Δ*f* values were higher than the targeted rate (1.0 or 0.1 %), particularly for the population with the smallest size (Tables 3, 4). For instance, for *N* = 20, a realised Δ*f* of about 2 % was obtained for both CHR__ove_ and REG__ove_ when the constraint was 1 %. This was also observed when the rate of coancestry was computed based on observed SNP genotypes.

**Table 4:** Average rate of genomic coancestry in genomic regions targeted for minimising coancestry and in the rest of the genome across generations (*t*) when applying different management strategies (CHR, CHR__ove_, REG, REG__ove_) for a population of size 100

*t*	CHR	CHR__ove_		REG	REG__ove_	
		*C* = 1.0 %	*C* = 0.1 %		*C* = 1.0 %	*C* = 0.1 %
Rate of genomic coancestry at regions targeted for minimisation (%)						
1	−4.69	−4.48	−4.18	−2.34	−1.93	−1.77
2	−1.83	−2.00	−1.94	−0.36	−0.40	−0.41
3	−0.81	−0.82	−0.86	−0.13	−0.17	−0.21
4	−0.67	−0.75	−0.91	−0.10	−0.07	0.17
5	−0.28	−0.36	−0.30	−0.10	0.10	0.17
10	−0.20	−0.17	−0.07	0.15	0.13	0.28
Rate of genomic coancestry at the rest of genome (%)						
1	3.43	1.45	0.67	1.74	1.30	0.47
2	1.70	1.44	0.65	1.21	1.16	0.45
3	2.17	1.26	0.34	1.14	1.12	0.46
4	1.32	1.35	0.52	1.03	1.05	0.43
5	2.02	1.23	0.45	1.00	1.03	0.45
10	1.44	1.16	0.42	0.98	0.89	0.42

Two different constraints (*C*) were imposed on the coancestry rate at the rest of the genome when applying strategies CHR__ove_ and REG__ove_

*CHR* contributions are optimised to minimise *f*_*m_chr*_, *CHR*_*_ove*_ contributions are optimised to minimise *f*_*m_chr*_ while restricting *f*_*m_ove*-*chr*_, *REG* contributions are optimised to minimise *f*_*m_reg*_, *REG*_*_ove*_ contributions are optimised to minimise *f*_*m_reg*_ while restricting *f*_*m_ove*-*reg*_

**Fig. 1:**
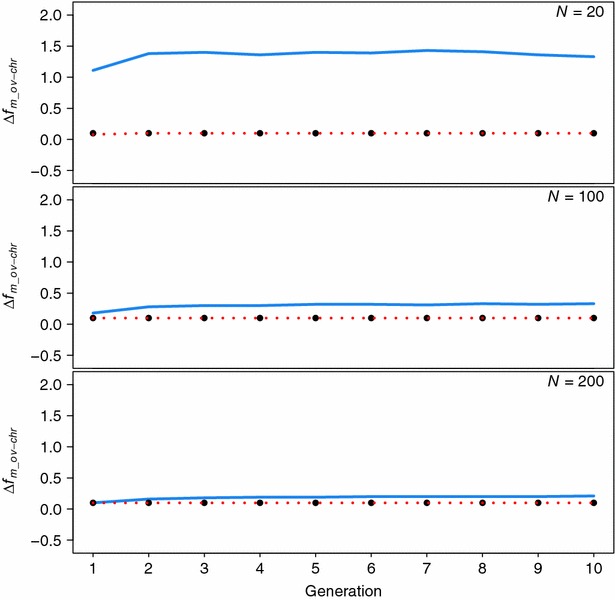
Expected (*dotted lines*) and observed (*straight lines*) rate of genomic coancestry computed for the whole genome except chromosome 1 (Δ*f*_*m_ove*-*chr*_, in %) in the offspring generation, when the optimisation strategy was CHR__ove_ with a restriction on the rate of coancestry in the rest of the genome of 0.1 % for three population sizes (*N*). The specific imposed restrictions are indicated as *filled circles*

In order to investigate if this unexpected result was a consequence of the optimisation process failing to find a solution that meets the imposed restriction, we calculated the expected rate of coancestry at *t* + 1 (E(Δ*f*_*t*+1_)) given the solutions from the optimisation and compared it with the actual rate observed after the offspring were generated. E(Δ*f*_*t*+1_) was calculated as (**c′**_*t*_ **G**_***x****t*_ **c**_*t*_ − *f*_*t*_)/(1 − *f*_*t*_), where **G**_***x***_ is the coancestry matrix of the candidates for the region in question and *f*_*t*_ the average coancestry in the candidates’ generation. Note that *f*_*t*_ and **G**_***x***_ were computed from marker genotypes. Figure 1 shows the expected and realised Δ*f*_*m_ove*-*chr*_ for a CHR__ove_ scheme for three populations of size 20, 100 and 200. Across all generations and population sizes, the expected rate always met the requirement of being lower than the imposed restriction, but the realised value was always higher than the restriction (i.e. the restriction was set at 0.1 %, but the realised rate across generations was approximately 1.4, 0.3 and 0.2 % for *N* = 20, 100 and 200, respectively). Figure 1 also shows that the differences between expected and realised Δ*f*_*m_ove*-*chr*_ tended to increase when *N* decreased.

**Fig. 2:**
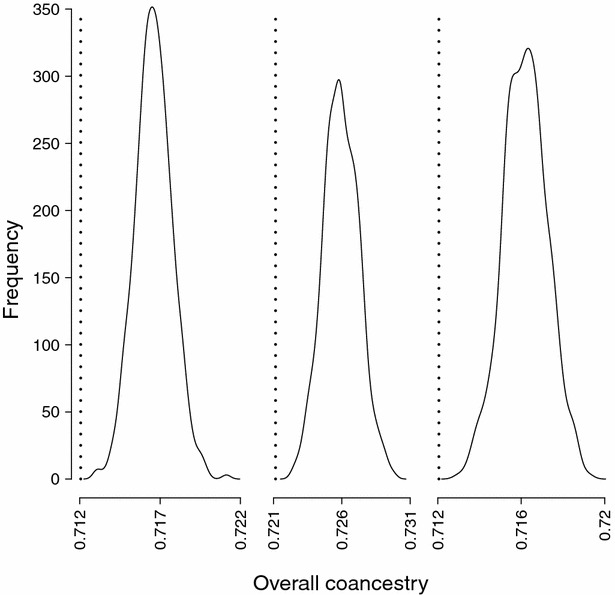
Distribution of observed average genomic coancestry in the offspring generation of three sets of parents. For each set of parents 1000 offspring generations were created using the same parental optimised contributions. The size of the assumed population was *N* = 20, and the optimisation strategy was CHR__ove_ with an overall coancestry restriction of 0.1 %. *Dotted lines* indicate the targeted coancestry for each set of parents

Although the results shown in Fig. 1 were consistent across replicates, generations and population sizes, another analysis was carried out in order to test if these results can be explained by variation due to Mendelian inheritance. Given a group of selected candidates which were assigned an optimal contribution to fulfil a given constraint, 1000 sets of offspring generations were created using the same contributions. For each set, the realised average coancestry for the restricted regions was calculated and compared to the expected value. Figure 2 shows the distribution of average coancestry for three independent sets of parents (and consequently, with different optimal contributions). For all three sets of parents, the realised average coancestry in the offspring generation was always higher than the expected value given the optimal contribution. Specifically, the observed rate of coancestry was approximately double the intended rate.

Table 5 shows the results for strategies OVE and OVE__reg_, for which contributions were optimised for minimising overall coancestry (*f*_*m_ove*_) with or without a restriction on the increase of coancestry at specific regions of the genome (Δ*f*_*m_reg*_). Here, the restrictions on Δ*f*_*m_reg*_ used in strategy OVE_reg were more stringent (i.e. *C* = 0.10 and 0.01 %) than those imposed under strategies CHR_ove and REG_ove (i.e. *C* = 1 and 0.1 %). The inclusion of a constraint on Δ*f*_*m_reg*_ (i.e. OVE__reg_) managed to lower Δ*f* (and such reduction was greater with the more stringent constraint, e.g., *C* = 0.01 %), but, as in CHR__ove_ and REG__ove_, the realised values did not fulfil the imposed constraint (i.e. the realised Δ*f*_*m_reg*_ was greater than the *C* used).

**Table 5:** Average rate of genomic coancestry (in %) in specific regions and in the rest of the genome across generations (*t*), when applying two different management strategies (OVE and OVE__reg_) for populations of two different sizes (*N*)

*N*	*t*	Specific regions			Rest of the genome		
		OVE	OVE__reg_		OVE	OVE__reg_	
			*C* = 0.10 %	*C* = 0.01 %		*C* = 0.10 %	*C* = 0.01 %
20	1	0.35	0.35	0.35	0.23	0.25	0.25
	2	1.23	0.81	0.80	1.07	1.05	1.09
	3	1.33	1.27	0.67	1.14	1.15	1.23
	4	0.89	1.23	0.83	1.08	1.15	1.05
	5	0.98	0.92	0.76	1.06	1.06	1.19
	10	0.81	0.68	0.84	1.07	1.07	1.16
100	1	−0.48	−0.49	−0.49	−0.51	−0.49	−0.47
	2	−0.15	−0.19	−0.16	−0.16	−0.11	−0.12
	3	−0.18	−0.15	−0.19	−0.07	−0.07	−0.06
	4	−0.05	−0.07	−0.05	−0.06	−0.09	−0.06
	5	−0.25	−0.27	−0.29	−0.03	−0.02	−0.07
	10	−0.05	−0.05	−0.02	−0.03	−0.03	−0.03

Two different constraints (*C*) were imposed on the coancestry rate in each of the specific regions when applying strategy OVE__reg_

*OVE* contributions are optimised to minimise *f*_*m_ove*_, *OVE*_*_reg*_ contributions are optimised to minimise *f*_*m*_*ove*_ while restricting Δ*f*_*m*_*reg*_

An interesting observation among these scenarios was the proportion of candidates which were selected to contribute offspring to the following generation and the variance of the number of offspring generated by each candidate (Tables 6, 7). Under random selection, this variance was close to 2, which is the theoretical expected value if contributions follow a Poisson distribution. The optimisation using pedigree coancestry resulted in all individuals contributing equally (i.e., every candidate generates two offspring), which is expected since all individuals in the base population were assumed to be non-inbred and unrelated. However, when considering genomic coancestry, the proportion of selected candidates differed substantially across strategies with OVE having the largest number of selected candidates, CHR the smallest and REG somewhere in between. In general, in the first generation, the proportion of candidates actually contributing was lower than in the following generations. This proportion was also lower for *N* = 100 than for *N* = 20. The inclusion of a constraint on the rate of coancestry in the minimisation (OVE__reg_, REG__ove_, CHR__ove_) resulted in a slight increase in the number of selected candidates (cf. OVE, CHR, REG).

**Table 6:** Percentage of individuals that contributed to the next generation (*N*_*cont*_) and variance of the number of offspring (*V*_*c*_) when applying different management strategies (RAN, PED, OVE, REG, CHR) for populations of two different sizes (*N*)

*N*	*t*	RAN		PED		OVE		REG		CHR	
		*N*_*cont*_	*V*_*c*_	*N*_*cont*_	*V*_*c*_	*N*_*cont*_	*V*_*c*_	*N*_*cont*_	*V*_*c*_	*N*_*cont*_	*V*_*c*_
20	0	88.4	1.77	100	0	81.0	2.28	56.7	6.00	47.0	8.57
	1	87.0	1.83	100	0	90.3	1.37	61.1	4.88	49.0	7.30
	2	88.4	1.80	100	0	91.4	1.25	62.2	4.63	53.8	6.22
	3	86.0	2.03	100	0	89.8	1.40	65.4	4.06	56.2	5.72
	4	88.5	1.70	100	0	90.1	1.33	65.5	3.98	56.7	5.61
	9	89.5	1.64	100	0	90.5	1.26	71.6	3.18	63.3	4.41
100	0	85.6	2.10	100	0	54.3	6.33	34.0	13.67	23.6	23.64
	1	86.4	1.97	100	0	60.1	5.12	38.4	11.49	26.3	18.46
	2	87.1	1.95	100	0	61.4	4.88	39.9	10.54	28.6	16.88
	3	86.1	2.07	100	0	61.3	4.52	42.0	9.88	29.1	15.49
	4	87.9	1.96	100	0	65.0	4.43	44.6	9.10	30.1	15.86
	9	86.9	1.99	100	0	81.0	2.28	51.3	6.97	47.0	8.57

*RAN* contributions are assigned at random, *PED* contributions are optimised to minimise *f*_*p*_, *OVE* contributions are optimised to minimise *f*_*m_ove*_, *REG* contributions are optimised to minimise *f*_*m_reg*_, *CHR* contributions are optimised to minimise *f*_*m_chr*_

**Table 7:** Percentage of individuals that contributed to the next generation (*N*_*cont*_) and variance of the number of offspring (*V*_*c*_) when applying different management strategies (OVE__reg_, REG__ove_, CHR__ove_) for populations of two different sizes (*N*)

*N*	*t*	OVE__reg_				REG__ove_				CHR__ove_			
		*C* = 0.10 %		*C* = 0.01 %		*C* = 1.0 %		*C* = 0.10 %		*C* = 1.0 %		*C* = 0.10 %	
		*N*_*cont*_	*V*_*c*_	*N*_*cont*_	*V*_*c*_	*N*_*cont*_	*V*_*c*_	*N*_*cont*_	*V*_*c*_	*N*_*cont*_	*V*_*c*_	*N*_*cont*_	*V*_*c*_
20	0	84.8	1.75	88.1	1.44	72.3	3.68	76.70	5.99	63.6	4.58	68.8	3.73
	1	93.1	1.04	96.2	0.78	80.6	2.14	81.10	4.88	69.8	3.55	79.1	2.52
	2	94.8	0.90	96.7	0.69	83.1	1.90	82.30	4.62	70.1	3.44	78.0	2.41
	3	94.4	0.92	96.6	0.70	84.1	1.81	85.20	4.07	72.9	3.24	81.2	2.24
	4	94.8	0.86	96.5	0.71	84.1	1.82	85.50	4.00	72.6	3.28	82.3	2.06
	9	95.1	0.85	97.4	0.68	85.9	1.78	89.70	3.17	73.4	3.03	83.6	1.91
100	0	55.4	6.12	56.0	5.83	38.1	11.48	51.90	7.12	35.5	13.29	43.9	9.59
	1	61.4	4.78	61.4	5.05	40.2	10.43	56.70	5.96	35.0	14.14	47.0	7.95
	2	63.8	4.43	64.6	4.15	40.9	10.28	57.80	5.47	36.1	12.74	49.6	7.70
	3	64.7	4.29	66.1	3.97	43.4	9.51	58.10	5.43	35.4	12.92	49.4	7.91
	4	65.8	4.08	66.5	4.04	44.0	9.08	60.60	5.00	35.5	13.42	51.8	7.21
	9	67.5	3.74	69.5	3.54	50.3	7.31	63.70	4.39	38.5	11.30	52.9	6.38

Different restrictions (*C*) were applied on the rate of coancestry

*OVE*_*_reg*_ contributions are optimised to minimise *f*_*m*_*ove*_ while restricting Δ*f*_*m*_*reg*_, *REG*_*_ove*_ contributions are optimised to minimise *f*_*m_reg*_ while restricting *f*_*m_ove*-*reg*_, *CHR*_*_ove*_ contributions are optimised to minimise *f*_*m_chr*_ while restricting *f*_*m_ove*-*chr*_

## Discussion

This study shows that the optimal contribution method based on semidefinite programming can use genomic coancestry calculated from dense panels of biallelic molecular markers to efficiently control the loss of genetic variability in specific genomic regions. Moreover, the method can also be easily extended to add constraints for simultaneously maintaining the loss of diversity across the rest of the genome at an acceptable rate. This study also found that the prediction of the expected level of coancestry in the offspring generation based on current genetic contribution theory can be biased downwards when using genomic coancestries. This may result in the rate of loss in genetic diversity at restricted regions being higher than that set during management.

Genomic relationship matrices that are based on high-density SNP genotyping data can more accurately reflect the true relationships between individuals than the standard pedigree-based coancestry matrix because they take into account the variability in the genetic information received by each full-sib due to Mendelian segregation of SNPs. Hence, it is not surprising that using the genomic coancestry matrix in OC (OVE) was more efficient in preserving genetic diversity than using pedigree-based relationships (PED). However, this was true only if the level of LD in the SNP panel was sufficiently high to represent the genome sequence not covered by SNPs. This study showed that the rate of average coancestry was better minimised under strategy OVE than under strategy PED for the population generated with the lowest mutation rate (which led to higher LD in the SNP panel). The superiority of OVE over PED was clear for *N* = 100, with the genetic diversity even increasing with OVE (Table 2). These results were, however, not reproduced when considering the population with the highest mutation rate. In this case, OVE performed better (i.e., lower Δ*f*) than PED in the first generation, but slightly worse (i.e., higher Δ*f*) in later generations. This finding agrees with previous results of Gómez-Romano et al. [8].

The use of genomic relationship matrices also allowed us to maintain (or even increase) genetic diversity in the targeted regions (Tables 3, 4). The efficiency in maintaining genetic diversity was greater when the regions were located on a single chromosome (CHR) than when they were scattered across different chromosomes (REG). This is not surprising since the level of coancestry in a region will be somewhat correlated to that of other regions on the same chromosome, thus a solution for maintaining variability may be equally good or bad for all regions on the same chromosome. This is consistent with the finding that the optimization was more efficient when it aimed at maintaining the genetic diversity in only a few regions than when the entire genome was targeted (cf. OVE vs. CHR and REG; Tables 2, 3, 4).

However, the efficiency in reducing the rate of coancestry in specific regions was accompanied by a substantial increase in the rate of coancestry across the rest of the genome (which could be several fold higher than when assuming random selection), as previously described by Roughsedge et al. [14]. Moreover, the performance in preserving genetic diversity in the targeted regions was paralleled by a detrimental effect observed across the rest of the genome (i.e., CHR preserved genetic diversity in the targeted regions more efficiently than REG, but it resulted in more loss of diversity for the rest of the genome). Our work shows that this undesired consequence can be mitigated by imposing a constraint on the rate of coancestry across the rest of the genome (cf. REG__ove_ and CHR__ove_; Tables 3, 4).

We observed an unexpected result when minimising the rate of coancestry for specific regions and simultaneously imposing a restriction on the rate of coancestry in other regions (strategies REG__ove_ and CHR__ove_, OVE__reg_), i.e. the realised rate of coancestry in the restricted regions was always higher than the imposed restriction, particularly for the lowest *N* value (Tables 3, 4, 5). This result was observed in spite of the fact that the optimal solution fulfilled the restriction that the expected Δ*f*_*m_ove*-*chr*_ should not be larger than the restriction imposed (Figs. 1, 2). This finding is similar to that previously reported by Roughsedge et al. [14] who also showed a clear discrepancy between the observed and expected rates of molecular inbreeding at specific positions of the genome. This leads to the conclusion that, when using genomic coancestry matrices, the equation *f* = **c′Gc** is a biased estimator of the expected mean coancestry in the next generation. This equation was adopted from the genetic contribution theory [17] and was derived based on the infinitesimal model and assuming that the coancestry matrix is calculated using pedigree information. Initially, it appeared justified to use this equation since the genomic relationship matrix is just a more refined estimate of the coancestry that accounts to some extent for the additional variation due to Mendelian inheritance. However, the fact that the expected value predicted with the equation was consistently biased downward and the magnitude of this bias depended on the population size (the smaller is *N*, the greater is the bias) suggests that some additional terms are arising from the Mendelian inheritance variance, which need to be accounted for when predicting the expected coancestry in the offspring generation. Hence, a revision of contribution theory is needed to properly use genomic relationship matrices to manage genetic diversity. This study showed that this biased predictor can still be used to control the change in coancestry but the amount of change may not be correctly estimated. However, this bias would have a more profound effect when using **c′Gc** as a restriction on the maximum coancestry to be allowed than when minimizing it in an objective function. Also from a practical point of view for breeding, not knowing the magnitude of the change (provided it is in the right direction) is probably less of a problem than crossing a threshold on the maximum rate allowed. Hence, refinement of the theory to account for this bias is more important for a breeding scheme where the objective is to maximise genetic gain while restricting the rate of coancestry to a given value. However, such breeding programmes generally involve populations of medium to large size, hence the expected bias with the current approach will be relatively small.

It is well known that in the absence of molecular or pedigree information, keeping equal numbers of males and females and constant census sizes (i.e., equalising contributions) is the most appropriate procedure to minimize loss of genetic diversity [18]. In the present study, pedigree relationships between individuals of the base population were assumed to be unknown (and individuals were assumed to be unrelated and non-inbred) and thus, when minimising Δ*f*_*p*_, the optimal solution was to equalise contributions. This occurred not only for the first generation but also in subsequent generations, provided the population remained homogeneous at the pedigree level. However, for strategies using genomic coancestry, equalising contributions was never the optimal solution because marker genotypes differentiated genetic relationships between pairs of individuals with the same degree of pedigree-based coancestry. In fact, the OVE strategy led to lower Δ*f* than the PED strategy, while using fewer individuals for breeding and with unequal contributions, especially for *N* = 100. This implies that, in addition to maintaining a higher level of genetic diversity, the use of genomic coancestry could have some economic advantages when managing genetic conservation programmes, since fewer animals need to be maintained (i.e., animals not contributing to the next generation could be discarded).

Several methods have been proposed and implemented in the past to solve the OC problem. They mainly fall into three different categories: (1) Lagrange multipliers [1, 2]; (2) genetic algorithms [19]; and (3) semidefinite programming (SDP) approaches [15]. The Lagrange multiplier approach is fast and very efficient but does not guarantee the optimal solution to be found [15]. Also, including additional constraints under the Lagrange multiplier approach requires major reformulation of the equations that are needed to find the optimal solution. Methodologies based on genetic algorithms are very flexible in terms of adding or removing constraints but the sampling approach on which the method is based means that optimality of the final solution cannot be verified. Also, they can be computer intensive, depending on the constraints included. The SDP approach guarantees that the solution found is the optimal. The method is also fast and flexible, since additional constraints can be easily added to the optimisation. In addition, general software packages for solving optimisation problems with SDP are available [16, 20–22].

The main limitation of the SDP methodology is that the constraints and objective functions need to be convex, which for the situation considered here means that the coancestry matrices must be positive definite. Such property should hold when the genomic relationship matrices are calculated using the method proposed by Nejati-Javaremi et al. [3], as done here. However, in practice, it is likely that genotypes will be missing for a proportion of the SNPs, so coancestries may be calculated with a slightly different set of SNPs for each pair of individuals, which in certain situations can result in the genomic relationship matrix being non-positive definite. This problem could be solved by adding a very small quantity to all diagonal elements of the matrix, so that it becomes positive definite. However, the consequences of this for optimality of the solution are yet to be quantified. Another potential problem is that the SDP implementation requires the inverse of the genomic relationship matrices (see “Appendix”), which may not exist, especially when considering small genomic regions. For instance, if two sibs inherit the same haplotypes for the region in question from their common parent, their relationship with the rest of the candidates will be the same and the resulting matrix will be non-invertible. Similarly, when the number of SNPs used to calculate the genomic relationship matrix is smaller than the number of candidates, the matrix will also be non-invertible. A solution for this problem could be to use the Moore–Penrose generalised inverse of the genomic matrix or to add a small constant to all diagonal elements. Further studies are required to determine the consequence of using generalised inverse matrices in this context.

A key component to successfully manage genetic diversity using genomic relationship matrices is that they are good estimates of the genomic coancestry, such that their use to predict the expected average coancestry in the offspring generation (i.e., *f* = **c′Gc**) is justified (although they may be biased). In this study, the allelic similarity method proposed by Nejati-Javaremi et al. [3] was used to calculate genomic relationships, since this method (i.e., $\left( {0.25} \right) \sum\nolimits_{i = 1}^{2} {\sum\nolimits_{j = 1}^{2} {\delta_{ij} } }$) has a ‘*natural’* interpretation in relation to the definition of the coefficient of coancestry (i.e., the probability of randomly sampling the same allele from both individuals). Other methods for calculating genomic relationships, mainly based on the cross-product of the centered (and normalised) genotype score, have also been proposed (e.g., [4]). For a given SNP, the relationship between two individuals *i* and *j*, is equal to (*x*_*i*_ − 2*p*)(*x*_*j*_ − 2*p*), where *x* is the number of reference alleles in the genotype (i.e., 0, 1, 2) and *p* is its frequency. Similarly, this relationship can be normalised (weighted) by its frequency, leading to an estimate equal to (*x*_*i*_ − 2*p*)(*x*_*j*_ − 2*p*)/(2*p*(1 − *p*)). Then, the overall relationship between two individuals is the average across all SNPs in the whole genome or in the regions of interest (times some constant). Such relationship matrices have been widely used with great success in different methods to calculate genomic predictions of breeding values (e.g. [5]), but the justification for using them as an estimate of the coefficient of coancestry (and thereby its validity to model genetic diversity) is less clear. First, a close examination of both (the normalised and the un-normalised) formulae shows that the values for the relationship estimates range from −1 to x, where x can be substantially larger than 1 (depending on the allele frequency). This means that relationship estimates calculated with these methods can be clearly outside the valid range for a coefficient of coancestry (i.e., [0:1]). Practical experience has shown that estimates of the average coancestry across the whole genome tend to be within the valid range but this may not be the case when considering a smaller region with few genotyped SNPs. Second, the relationship calculated from the cross-product of the centered genotype score results in a pair of individuals that are homozygous for the minor allele in having a higher relationship value than another pair of individuals that are homozygous for the most frequent allele and, thus, those carrying rare alleles would be penalised when optimising contributions. Third, centering the genotype score makes the matrix non-invertible (even when the number of SNPs in the region is larger than the number of candidates), which adds a complication to the semidefinite optimisation.

However, it is conceivable that, in practical conservation programmes, one may need to consider both methods to calculate genomic relationship matrices (i.e. allelic similarity and crossproduct of genotype score), depending on what is needed for the preserved population. The use of these genomic relationship matrices in the objective function impacts the trajectory of the change in gene frequency in different manners. Intuitively, it appears that optimisation with the ‘allelic-similarity’ matrices [3] would favour solutions that tend to drive the gene frequency towards 0.5. Conversely, optimisation using the ‘crossproduct’ matrices [4] will lead to solutions that are closer to the average gene frequency, and thus will attempt to keep the gene frequency unchanged (although rare alleles may still be lost due to drift). Thus, considering that conservation programmes generally aim at (1) increasing genetic diversity and (2) maintaining the uniqueness of the population, the choice of how coancestry is quantified may depend on which of these two objectives is more important. On the one hand, using the ‘allelic similarity’ matrix may increase genetic diversity but also the risk of changing the characteristics of the population. However, on the other hand, use of the ‘cross-product’ matrix will favour the *status quo,* i.e. will better preserve the original characteristics of the population but at the same time, rare alleles may be lost due to drift. Clearly, a more extensive study is necessary to understand the principles that justify the use of different genomic relationship matrices in management of genetic diversity.

In this study, we developed OC methodology to separately control genetic diversity in specific regions of the genome, and thereby allow for a better and more customised solution to management of genetic diversity. This added flexibility has great value since genomic data shows that nucleotide diversity varies greatly across the genome (e.g., [12, 13]), probably as a result of evolutionary forces such as genetic drift and selection. In practical conservation programmes, regions that display a greater loss of diversity should be prioritized to better avoid further loss of diversity in those regions. Consequently, the conservation scheme would be more successful by putting more emphasis on these regions (using schemes such as REG_reg or OVER_reg), rather than just maximising the overall average diversity. Moreover, there are genomic regions that include specific loci that are particularly relevant in terms of genetic diversity and it would be useful to be able to control these regions separately. Examples are the MHC region that is involved in general resistance to disease and regions that have been reported to be responsible for inbreeding depression [9, 10, 23]. Clearly, a high level of genetic diversity is desirable in those regions to ensure that the population can deal with potential new disease challenges and to avoid the detrimental effects of inbreeding depression. The list of regions that include genes of interest is not complete (and probably never will be) but it is likely to increase over time. Thus, breeding programmes should begin by considering a few regions and gradually become more sophisticated by adding more (with newly discovered loci), while perhaps reducing the length of each region. Clearly, the approach proposed here will permit a much better control in the management of genetic diversity in conservation programmes.

## Conclusions

This study confirms that the use of genomic coancestry in the optimisation of contributions is substantially more efficient in maintaining genetic diversity than the use of pedigree coancestry. Moreover, the use of genomic coancestry permits the targeting of specific genomic regions to minimise the loss of genetic diversity and the extension of the optimisation procedure to include restrictions for additional regions. This study also highlighted the need to refine the theory of genetic contributions using realised genomic relationship matrices in order to ensure that optimal contribution methods properly manage the genetic diversity available in a population.

## Appendix: Formulation of the optimisation of genetic contributions to minimise loss of diversity as a standard semidefinite programming

The optimisation of genetic contributions to minimise the increase of average coancestry (i.e., the loss of genetic diversity) is reformulated as a standard semidefinite programming using the same approach that was proposed by Pong-Wong and Woolliams [15]. However, small variations in the definition of genetic relationships and refinements in the optimisation problem mean that equations representing the standard semidefinite programming are slightly different to those reported by Pong-Wong and Woolliams [15]. The purpose of this Appendix is to briefly describe the precise reformulation of the optimisation problem used in this study.

Pong-Wong and Woolliams [15] showed that the problem of optimisation of contribution can be reformulated as a standard semidefinite programming, and thereby solved using such an approach. Following the notation of Vandenberghe and Boyd [24], the standard form for a semidefinite programming problem is:

$$ {\text{Minimise}}\quad {\mathbf{a}}^{\prime }{\mathbf{x}}, $$

$$ {\text{subject to}}\quad {\mathbf{Y}} \ge \textbf{0}, {\mathbf{Y}} = {\mathbf{Y}}_{0} + \mathop \sum \limits_{{{\mathbf{i}} = 1}}^{{\mathbf{n}}} {\mathbf{Y}}_{{\mathbf{i}}} {\text{x}}_{{\mathbf{i}}} , $$

where **a** is the vector of ‘cost’, **x** is the vector of *n* variables to be optimised, *x*_*i*_ is the *i* element of **x**, **Y** is a positive semidefinite matrix with *n* + 1 affine matrices (**Y**_*i*_, *i* = 0, 1, 2, …, *n*). The matrix inequality **Y** ≥ 0 means that **Y** is positive semidefinite.

### Optimisation problem 1

Following the same approach as Pong-Wong and Woolliams [15], the reformulation of optimisation problem 1 is done by (1) introducing an auxiliary variable *v* to serve as the upper limit of the objective function, (2) using the Shur complement to give a linear expression to the quadratic constraint; and (3) replacing the equality constraints by inequality constraints. Hence, the optimisation problem 1 is reformulated as the optimisation of *v* and **c** to:

$$ {\text{Minimise}}\quad v, $$

$$ \begin{aligned} {\text{subject to}}\quad \left[ {\begin{array}{*{20}c} {{\mathbf{G}}^{ - 1} } & {\mathbf{c}} \\ {\mathbf{c}} & v \\ \end{array} } \right] \ge 0, \hfill \\ {\mathbf{c}}^{\prime }{\mathbf{s}} - 0. 5 { } \ge \, 0, \hfill \\ - {\mathbf{c}}^{\prime }{\mathbf{s}} + 0. 5 { } \ge \, 0, \hfill \\ {\mathbf{c}}^{\prime }{\mathbf{d}} - 0. 5 { } \ge \, 0, \hfill \\ - {\mathbf{c}}^{\prime }{\mathbf{d}} + 0. 5 { } \ge \, 0, \hfill \\ {\mathbf{c}} \ge \, 0. \hfill \\ \end{aligned} $$

Then, matrix **Y** accounting for the six constraints is a block diagonal matrix of the form:

$$ \varvec{Y} = \left[ {\begin{array}{*{20}c} {\left[ {\begin{array}{*{20}c} {{\mathbf{G}}^{ - 1} } & {\mathbf{c}} \\ {{\mathbf{c}}^{\prime }} & v \\ \end{array} } \right]} & {} & {} & {} & {} & {} \\ {} & {\left[ {{\mathbf{c}}^{\prime }{\mathbf{s}} - 0.5} \right]  } & {} & {} & {} & {} \\ {} & {} & {\left[ { - {\mathbf{c}}^{\prime }{\mathbf{s}} + 0.5} \right]} & {} & {} & {} \\ {} & {} & {} & {\left[ {{\mathbf{c}}^{\prime }{\mathbf{d}} - 0.5} \right]} & {} & {} \\ {} & {} & {} & {} & {\left[ { - {\mathbf{c}}{^\prime }{\mathbf{d}} - 0.5} \right]} & {} \\ {} & {} & {} & {} & {} & {\left[ {{\text{diag}}({\mathbf{c}})} \right]} \\ \end{array} } \right] $$

with the (*n* + 2) affine matrices of **Y** equal to:

$$ {\mathbf{Y}}_{0} = \left[ {\begin{array}{*{20}c} {\left[ {\begin{array}{*{20}c} {{\mathbf{G}}^{ - 1} } & {\textbf{0}_{{(\varvec{nx}1)}} } \\ {\textbf{0}_{{(1\varvec{xn})}} } & 0 \\ \end{array} } \right]} & {} & {} & {} & {} & {} \\ {} & { - 0.5  } & {} & {} & {} & {} \\ {} & {} & {0.5} & {} & {} & {} \\ {} & {} & {} & { - 0.5} & {} & {} \\ {} & {} & {} & {} & {0.5} & {} \\ {} & {} & {} & {} & {} & {\left[ \textbf{0} \right]} \\ \end{array} } \right] $$

$$ {\mathbf{Y}}_{\varvec{i}} = \left[ {\begin{array}{*{20}c} {\left[ {\begin{array}{*{20}c} {0_{{(\varvec{nxn})}} } & {{\mathbf{I}}_{\varvec{i}} } \\ {{\mathbf{I}}_{\varvec{i}}^{\varvec{'}} } & 0 \\ \end{array} } \right]} & {} & {} & {} & {} & {} \\ {} & {{\mathbf{s}}_{i}  } & {} & {} & {} & {} \\ {} & {} & { - {\mathbf{s}}_{\text{i}} } & {} & {} & {} \\ {} & {} & {} & {{\mathbf{d}}_{i} } & {} & {} \\ {} & {} & {} & {} & { - {\mathbf{d}}_{i} } & {} \\ {} & {} & {} & {} & {} & {\left[ {{\mathbf{diag}}({\mathbf{I}}_{\varvec{i}} )} \right]} \\ \end{array} } \right], \quad i = 1,n, $$

and

$$ {\mathbf{Y}}_{{\varvec{n} + 1}} = \left[ {\begin{array}{*{20}c} {\left[ {\begin{array}{*{20}c} {\textbf{0}_{{(\varvec{nxn})}} } & {\textbf{0}_{{(\varvec{nx}1)}} } \\ {\textbf{0}_{{(1\varvec{xn})}} } & 1 \\ \end{array} } \right]} & {} & {} & {} & {} & {} \\ {} & 0 & {} & {} & {} & {} \\ {} & {} & 0 & {} & {} & {} \\ {} & {} & {} & 0 & {} & {} \\ {} & {} & {} & {} & 0 & {} \\ {} & {} & {} & {} & {} & {\left[ {\textbf{0}_{{(\varvec{nxn})}} } \right]} \\ \end{array} } \right], $$

where the size of the first block is (*n* + 1) × (*n* + 1), the sizes of the next four are 1 × 1 and the size of the last one is *n* × *n*. **0**_*(j*×*k)*_ are matrices/vectors of zeros of size *j* × *k*, **I**_*i*_ is the *i* column of the identity matrix of size *n* × *n* and **diag**(**I**_*i*_) is a diagonal matrix with diagonal equal to **I**_*i*_. All elements outside the block diagonal matrices are 0. Note that the formulation described above differs from that given in Eq. (8) of Pong-Wong and Woolliams [15] by a constant value in first block of **Y**_**0**_. This is to account for the difference in the definition of relationship matrix (i.e., here the relation matrix contains coefficients of coancestry between individuals; whereas it is twice this value for Pong-Wong and Woolliams [15]).

### Optimisation problem 2

Reformulation of optimisation problem 2 is similar to that above, but the *m* additional constraints need to be added. Hence, using the Shur complement again, the formulation of (2) becomes:

$$ {\text{Minimise}}\quad v, $$

$$ {\text{subject to}}: $$

$$ \left[ {\begin{array}{*{20}c} {{\mathbf{G}}^{ - 1} } & {\mathbf{c}} \\ {\mathbf{c}} & v \\ \end{array} } \right] \ge 0, $$

$$ \begin{array}{l} \left[ {\begin{array}{*{20}c} {{\mathbf{G}}_{\varvec{j}}^{ - 1} } & {\mathbf{c}} \\ {\mathbf{c}} & {k_{j} } \\ \end{array} } \right] \ge 0,\, j = 1, m \hfill \\ {\mathbf{c}}^{\prime }{\mathbf{s}} - 0. 5 { } \ge \, 0, \hfill \\ - {\mathbf{c}}^{\prime }{\mathbf{s}} + 0. 5 { } \ge \, 0, \hfill \\ {\mathbf{c}}^{\prime }{\mathbf{d}} - 0. 5 { } \ge \, 0, \hfill \\ - {\mathbf{c}}^{\prime }{\mathbf{d}} + 0. 5 { } \ge \, 0, \hfill \\ {\mathbf{c}} \ge \, 0, \hfill \\ \end{array} $$

and the matrix **Y** is augmented to be:

$$ \varvec{Y} = \left[ {\begin{array}{*{20}c} {\left[ {\begin{array}{*{20}c} {{\mathbf{G}}^{ - 1} } & {\mathbf{c}} \\ {{\mathbf{c}}'} & v \\ \end{array} } \right]} & {} & {} & {} & {} & {} & {} \\ {} & {\left[ {\begin{array}{*{20}c} {\left[ {\begin{array}{*{20}c} {{\mathbf{G}}_{1}^{ - 1} } & {\mathbf{c}} \\ {{\mathbf{c}}^{\prime }} & {k_{1} } \\ \end{array} } \right]} & \cdots & {} \\ \vdots & \ddots & \vdots \\ {} & \cdots & {\left[ {\begin{array}{*{20}c} {{\mathbf{G}}_{\varvec{j}}^{ - 1} } & {\mathbf{c}} \\ {{\mathbf{c}}^{\prime }} & {k_{j} } \\ \end{array} } \right]} \\ \end{array} } \right]} & {} & {} & {} & {} & {} \\ {} & {} & {\left[ {{\mathbf{c}}^{\prime }{\mathbf{s}} - 0.5} \right]  } & {} & {} & {} & {} \\ {} & {} & {} & {\left[ { - {\mathbf{c}}^{\prime }{\mathbf{s}} + 0.5} \right]} & {} & {} & {} \\ {} & {} & {} & {} & {\left[ {{\mathbf{c}}^{\prime }{\mathbf{d}} - 0.5} \right]} & {} & {} \\ {} & {} & {} & {} & {} & {\left[ { - {\mathbf{c}}^{\prime }{\mathbf{d}} - 0.5} \right]} & {} \\ {} & {} & {} & {} & {} & {} & {\left[ {{\text{diag}}({\mathbf{c}})} \right]} \\ \end{array} } \right], $$

with the (*n* + 2) affine matrices of **Y** equal to:

$$ {\mathbf{Y}}_{0} = \left[ {\begin{array}{*{20}c} {\left[ {\begin{array}{*{20}c} {{\mathbf{G}}^{ - 1} } & {\textbf{0}_{{(\varvec{nx}1)}} } \\ {\textbf{0}_{{(1\varvec{xn})}} } & 0 \\ \end{array} } \right]} & {} & {} & {} & {} & {} & {} \\ {} & {\left[ {\begin{array}{*{20}c} {\left[ {\begin{array}{*{20}c} {{\mathbf{G}}_{1}^{ - 1} } & {\textbf{0}_{{(\varvec{nx}1)}} } \\ {\textbf{0}_{{(1\varvec{xn})}} } & {K_{1} } \\ \end{array} } \right]} & \cdots & {} \\ \vdots & \ddots & \vdots \\ {} & \cdots & {\left[ {\begin{array}{*{20}c} {{\mathbf{G}}_{\varvec{j}}^{ - 1} } & {\textbf{0}_{{(\varvec{nx}1)}} } \\ {\textbf{0}_{{(1\varvec{xn})}} } & {k_{j} } \\ \end{array} } \right]} \\ \end{array} } \right]} & {} & {} & {} & {} & {} \\ {} & {} & { - 0.5  } & {} & {} & {} & {} \\ {} & {} & {} & {0.5} & {} & {} & {} \\ {} & {} & {} & {} & { - 0.5} & {} & {} \\ {} & {} & {} & {} & {} & {0.5} & {} \\ {} & {} & {} & {} & {} & {} & {\left[ \textbf{0} \right]} \\ \end{array} } \right], $$

$$ {\mathbf{Y}}_{\varvec{i}} = \left[ {\begin{array}{*{20}c} {\left[ {\begin{array}{*{20}c} {\textbf{0}_{{(\varvec{nxn})}} } & {{\mathbf{I}}_{\varvec{i}} } \\ {{\mathbf{I}}_{\varvec{i}}^{\varvec{'}} } & 0 \\ \end{array} } \right]} & {} & {} & {} & {} & {} & {} \\ {} & {\left[ {\begin{array}{*{20}c} {\left[ {\begin{array}{*{20}c} {\textbf{0}_{{(\varvec{nxn})}} } & {{\mathbf{I}}_{\varvec{i}} } \\ {{\mathbf{I}}_{\varvec{i}}^{\varvec{'}} } & 0 \\ \end{array} } \right]} & \cdots & {} \\ \vdots & \ddots & \vdots \\ {} & \cdots & {\left[ {\begin{array}{*{20}c} {\textbf{0}_{{(\varvec{nxn})}} } & {{\mathbf{I}}_{\varvec{i}} } \\ {{\mathbf{I}}_{\varvec{i}}^{\varvec{'}} } & 0 \\ \end{array} } \right]} \\ \end{array} } \right]} & {} & {} & {} & {} & {} \\ {} & {} & {{\mathbf{s}}_{i}  } & {} & {} & {} & {} \\ {} & {} & {} & { - {\mathbf{s}}_{i} } & {} & {} & {} \\ {} & {} & {} & {} & {{\mathbf{d}}_{i} } & {} & {} \\ {} & {} & {} & {} & {} & { - {\mathbf{d}}_{i} } & {} \\ {} & {} & {} & {} & {} & {} & {\left[ {{\mathbf{diag}}({\mathbf{I}}_{\varvec{i}} )} \right]} \\ \end{array} } \right] ,\, i = 1,n, $$

and

$$ {\mathbf{Y}}_{{\varvec{n} + 1}} = \left[ {\begin{array}{*{20}c} {\left[ {\begin{array}{*{20}c} {\textbf{0}_{{(\varvec{nxn})}} } & {\textbf{0}_{{(\varvec{nx}1)}} } \\ {\textbf{0}_{{(1\varvec{xn})}} } & 1 \\ \end{array} } \right]} & {} & {} & {} & {} & {} & {} \\ {} & {\left[ {\begin{array}{*{20}c} {\left[ {\begin{array}{*{20}c} {\textbf{0}_{{(\varvec{nxn})}} } & {\textbf{0}_{{(\varvec{nx}1)}} } \\ {\textbf{0}_{{(1\varvec{xn})}} } & 0 \\ \end{array} } \right]} & \cdots & {} \\ \vdots & \ddots & \vdots \\ {} & \cdots & {\left[ {\begin{array}{*{20}c} {\textbf{0}_{{(\varvec{nxn})}} } & {\textbf{0}_{{(\varvec{nx}1)}} } \\ {\textbf{0}_{{(1\varvec{xn})}} } & 0 \\ \end{array} } \right]} \\ \end{array} } \right]} & {} & {} & {} & {} & {} \\ {} & {} & 0 & {} & {} & {} & {} \\ {} & {} & {} & 0 & {} & {} & {} \\ {} & {} & {} & {} & 0 & {} & {} \\ {} & {} & {} & {} & {} & 0 & {} \\ {} & {} & {} & {} & {} & {} & {\left[ {\textbf{0}_{{(\varvec{nxn})}} } \right]} \\ \end{array} } \right]. $$

Once the optimisation has been reformulated as a standard semidefinite programming problem, it can easily be solved using general available purpose programmes.

In this study, the software SDPA was used to solve the optimisation problem. It is important to note that there is a slight difference between the definition for **Y** used here (i.e., ${\mathbf{Y}} = {\mathbf{Y}}_{0} + \sum\nolimits_{{\varvec{i} = 1}}^{\varvec{n}} {{\mathbf{Y}}_{\varvec{i}} x_{\varvec{i}} }$, adopted from Vandenbergh and Boyd [24]) and that used in SDPA (i.e., ${\mathbf{Y}} = - {\mathbf{Y}}_{0} + \sum\nolimits_{{\varvec{i} = 1}}^{\varvec{n}} {{\mathbf{Y}}_{\varvec{i}} x_{\varvec{i}} }$, [16]). Hence, from a practical point of view, the matrix **Y**_**0**_ described above needs to be multiplied by −1, before it is given as input to the SDPA programme.
